# Sexual competition and the evolution of condition‐dependent ageing

**DOI:** 10.1002/evl3.36

**Published:** 2018-01-13

**Authors:** Amy K. Hooper, Jussi Lehtonen, Lisa E. Schwanz, Russell Bonduriansky

**Affiliations:** ^1^ School of Biological, Earth and Environmental Sciences, Evolution and Ecology Research Centre University of New South Wales Sydney NSW 2052 Australia

**Keywords:** Ageing, condition‐dependence, resource acquisition, resource allocation, secondary sexual traits, sexual selection

## Abstract

Increased individual resources (condition) can be correlated with either increased or decreased longevity. While variation in resource acquisition and allocation can account for some of this variation, the general conditions that select for either pattern remain unclear. Previous models suggest that nonlinearity of payoffs from investment in reproduction (e.g., male secondary sexual traits) can select for high‐condition individuals that sacrifice longevity to increase reproductive opportunity. However, it remains unclear what mating systems or patterns of sexual competition might select for such life‐history strategies. We used a model of condition‐dependent investment to explore how expected payoffs from increased expression of secondary sexual traits affect optimal investment in lifespan. We find that nonlinearity of these payoffs results in a negative relationship between condition and lifespan under two general conditions: first, when there are accelerating marginal benefits from increasing investment; second, when individuals that invest minimally in secondary sexual trait expression can still achieve matings. In the second scenario, the negative relationship occurs due to selection on low‐condition individuals to extend lifespan at the cost of secondary sexual trait expression. Our findings clarify the potential role of sexual selection in shaping patterns of condition‐dependent ageing, and highlight the importance of considering the strategies of both low‐ and high‐condition individuals when investigating patterns of condition‐dependent ageing.

**Impact Summary**

In some species, when access to resources is increased, for example through higher quality diet, expected lifespan increases. Yet, in other species expected lifespan is decreased with increasing resources. Why do these different patterns exist? We developed a new theory to explore the conditions under which either pattern might occur. We uncovered two important factors related to investment in sexual traits (e.g., weapons, ornaments, or other traits that males use to compete for access to females and fertilizations) that might explain these different patterns of ageing. First, how mating success is determined by sexual trait expression is important. For example, if the highest quality males with the most elaborate sexual traits are able to effectively monopolize access to females, then the best strategy is to increase investment in those traits at the sacrifice of lifespan. On the other hand, if this type of monopolization does not occur then the best strategy for high quality males is to increase lifetime mating success by extending lifespan instead. Second, the ability to obtain matings without bearing elaborate traits is important. For example, a poor quality male may never have the resources to compete with high quality males. So, the best strategy may be to extend lifespan and rely on sneak matings to increase lifetime reproductive success. But this strategy can only evolve where sneak matings are possible. Both these factors can independently contribute to patterns ageing in response to individual access to resources. Our results show how the interplay between resources and mating systems could drive the evolution of different ageing strategies. This can shed light on broad patterns of ageing observed in the animal kingdom.

Ageing, the deterioration in performance with age, is an almost universal trait with important fitness consequences (Hughes and Reynolds 2005; Bouwhuis et al. 2012; Jones et al. 2014; Kowald and Kirkwood 2015), but the rate of ageing can vary considerably both between and within populations. There is growing evidence that some of this variation is related to variation in individual condition. We use the term “condition” here to refer to the pool of metabolic resources available to the individual to invest in all fitness‐enhancing traits. The size of this pool is determined by both environmental quality and individual genetic quality, and (at least under natural conditions, where resource overabundance is unlikely) an individual's condition is generally expected to influence the expression of its costly fitness‐enhancing traits and to predict its net fitness (Andersson 1982; Nur and Hasson 1984; Rowe and Houle 1996; Hill 2011). In males, condition‐dependent investment in reproduction via secondary sexual trait expression is well documented (Cotton et al. 2004; Bonduriansky 2007), as is the trade‐off between investment in reproduction and survival (Lemaître et al. 2015). Hence, variation in condition‐dependent ageing may be linked to condition‐dependent investment in secondary sexual traits.

Empirical investigations have revealed contrasting patterns of covariation between condition, secondary sexual trait expression, and ageing. In some species, high‐condition individuals have both increased expression of secondary sexual traits, and the longest lifespans. This includes crickets (Judge et al. 2008), damselflies (Munguía‐Steyer et al. 2010), zebra finches (Simons et al. 2012), scarlet‐tufted malachite sunbirds (Evans 2003), and barn swallows (Saino et al. 1997). Furthermore, overall reproductive success or fitness is positively correlated with lifespan in many species, such as mute swans (McCleery et al. 2008), great tits (Bouwhuis et al. 2009), and bighorn sheep (Bérubé et al. 1999), indicating that high‐condition individuals invest more in somatic maintenance and reproduction simultaneously.

However, a number of studies also show the opposite pattern, whereby high‐condition individuals experience accelerated ageing compared to low‐condition individuals. For example, male Neriid flies reared on a nutrient‐rich larval diet express exaggerated secondary sexual traits but experience faster reproductive ageing and decreased life spans (Hooper et al. 2017). Similar patterns have also been shown in bulb mites (Radwan and Bogacz 2000) and crickets (Hunt et al. 2004). Additionally, there is correlational evidence of this pattern in houbara bustards, where the most extravagant males experienced more rapid reproductive senescence (Preston et al. 2011).

Within‐species variation in condition‐dependent ageing also extends to differences between sexes. For example, in barn swallows, increasing tail length is associated with decreased male survival but with increased female survival (Møller and Szép 2002). Similarly, in Neriid flies, increased condition extends female lifespan, but decreases male lifespan (F. Spagopoulou, A. Hooper, Z. Wylde, R. Bonduriansky and A. A. Maklakov, unpubl. data). Interestingly, we have been unable to find examples of decreased lifespans in high‐condition females suggesting that the relationship between condition and ageing is more variable in males. Why the relationship between condition and ageing varies so markedly between and within species remains unclear.

Any resources invested in secondary sexual traits reduce resources that otherwise might be invested in traits such as intracellular machinery that maintains DNA integrity. This direct trade‐off between investment in reproduction and traits that maintain the soma is the basis for the disposable soma theory of ageing (Kirkwood 1977; Kirkwood and Rose 1991). A number of theoretical studies have shown that variation in this direct trade off and its relationship to variation in resource availability is central to understanding of variation in life history and ageing (van Noordwijk and de Jong 1986; Roff and Fairbairn 2007; Boggs 2009). Some studies have investigated the effect of resource availability on the shape of the trade‐off between investment in survival versus investment in reproduction (Descamps et al. 2016), while others have examined the timing of terminal investment strategies (Davison et al. 2014), effects of maternal age at breeding (van den Heuvel et al. 2016), and effects of compensatory growth (Mangel and Munsch 2005). These studies show that factors that promote unequal reproductive investment in low‐ versus high‐condition individuals can thereby affect patterns of condition‐dependent ageing. However, there have been few attempts to incorporate sexual selection into models of the evolution of ageing.

An important exception are models by Kokko, showing that optimal patterns of condition‐dependent investment may be determined by the shape of the payoff functions from investment in somatic maintenance versus investment in secondary sexual traits (Kokko 1998, 2001). In particular, Kokko (2001) showed that variation in the relationship between sexual advertisement and fitness returns can result in different relationships between condition and lifespan. When there are accelerating marginal fitness benefits from increasing investment in reproduction, this can select for high condition individuals to decrease investment in survival, resulting in a negative correlation between condition and survival. These models showed that both positive and negative relationships between condition and lifespan are possible in the context of the good genes hypothesis and honest signaling. However, the specific mating system parameters and patterns of sexual competition that select for different patterns of condition‐dependent ageing are still unclear.

Here, we extend these models to explore how variation in the payoff function for investment in secondary sexual traits can affect patterns of optimal investment in lifespan (via resource allocation to somatic maintenance) in individuals of varying condition. Kokko (2001) explored multiple functions representing payoffs from investment in advertisement (mating success), while always assuming a saturating function for the fitness payoffs from investment in survival. We extend Kokko's analysis to include functions where the fitness payoffs from investing in both survival and mating success were potentially variable. Furthermore, while Kokko showed that increasing nonlinearity of payoffs from investment in secondary sexual traits can select for high‐condition individuals to sacrifice investment in longevity, we ask whether this finding is generalizable to different function shapes representing other patterns of sexual competition, such as mating systems where low‐condition males engage in alternative reproductive tactics that do not require secondary sexual trait expression. We also model a wider range of condition values to encompass individuals that are extremely resource limited.

While our results support the findings of Kokko (2001), we also uncover another scenario that can select for a negative relationship between condition and lifespan. When low‐condition males are able to gain some mating success without investing in a secondary sexual trait, their investment strategy changes, and they are selected to increase investment in longevity. Hence, alternative tactics pursued by low‐condition individuals can shape patterns of condition‐dependent ageing.

## THE MODEL

As we are primarily interested in how secondary sexual trait expression affects mating success and ultimately fitness, our model focuses on investment decisions by males. Thus, in the context of the model description, “individuals” refers to males within a population.

We assume that all individuals in a population vary in the total amount of resources available to them, *T*, to invest in traits relating to fitness. *T* therefore represents individual condition (see Table 1 for all parameters). Individuals use this pool to invest in two traits related to fitness: somatic maintenance, *ω*, and a secondary sexual trait, *γ*. As we are primarily interested in investment in secondary sexual traits, we assume investment in γ is the only determinant of reproductive success. However, in many species reproductive success can depend on multiple traits, and investment in one reproductive trait can trade off against investment in another (e.g., Simmons et al. 2017). In such cases, γ can be interpreted as total reproductive investment (i.e., the sum of investments in all reproductive traits).

**Table 1:** Key terms and parameters

Term	Description
*T*	Total resources that the individual has available to allocate to fitness‐related traits
*ω*	Total investment in somatic maintenance
*γ*	Total investment in a secondary sexual trait
*q*	Proportion of *T* allocated to somatic maintenance
*W*	Total fitness
*b*	Mating success
*E*	Lifespan
q^*^	Value of *q* that optimises total fitness, *W*, for any given value of *T*
E^*^	Value of *E* that optimizes total fitness, *W*, for any given value of *T*
γ^*^	Value of *γ* that optimizes total fitness, *W*, for any given value of *T*
*p*	Lifespan function shape parameter
*n*	Power parameter for the power mating success function, which determines the shape of the relationship between *γ* and *b* as either linear (*n* = 1), decelerating power (0 < *n* < 1), or accelerating power (*n* > 1)
*m, r*	Parameters that determine the shape of the tan *b* function
*x, y, z*	Parameters that determine, respectively, the maximum, slope, and midpoint of the logistic *b* function
*s*	Determines minimum mating success for each *b* function

The sum of investment in ω and γ is limited by the individual's total resource pool (i.e., its condition), so that:

(1) T=ω+γ

Therefore, if *q* is the proportion of *T* that is invested in somatic maintenance, then:

(2) ω=qT

(3) $$\gamma \;=\left(1-q\right)\;T$$

Mating success, *b*, is determined by investment in the secondary sexual trait, and lifespan, *E*, is determined by investment in somatic maintenance, so that *b* is a function of *γ*, *E* is a function of *ω*, and hence both are functions of *q*. Individual fitness, *W*, is the product of mating success, *b*, and lifespan, *E*.

(4) W=bE

Hence, fitness is a function of secondary sexual trait expression and somatic maintenance, and individuals should invest more in whichever trait yields the higher marginal fitness returns on that investment. Below, we first find optimal *q*, denoted q^*^, which is the allocation to somatic maintenance that maximizes *W* for each value of condition over the range 0 < *T* < 1. This allows us to investigate the relationship between *T* and optimal lifespan, *E* ^*^, the lifespan that maximizes *W*.

**Figure 1:**
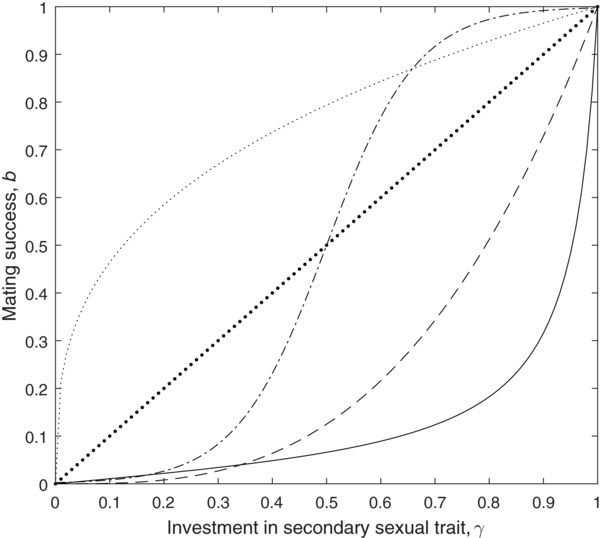
The five potential relationships for *b*(γ) explored in this article: Linear (dotted—large), decelerating power (dotted—small), accelerating power (dashed), and logistic (dash—dot), and tan (solid).

As mating success depends largely on external factors, including behaviors of other individuals, the relationship between secondary sexual trait expression and mating success can be expected to vary between populations, and take on a variety of function forms. As such, we investigate four potential relationships: Linear, decelerating power, accelerating power, and logistic (Fig. 1). We also included a tan function as a second accelerating power function, as was used in Kokko (2001). These functions will be influenced by an organism's ecology and mating system. For example, if only males with very exaggerated trait sizes achieve high mating success, then mating success will increase nonlinearly with increasing secondary sexual trait size, and can be described using a power function with accelerating returns, or, in the most extreme cases, by a tan function. Such relationships might be observed in species where males congregate in leks or defend large harems of females, such as in red deer (Kruuk et al. 2002). These two functions therefore represent similar biological scenarios, but in the tan function, the marginal benefits increase at an accelerating rate, and hence increase more steeply than in the accelerating power function. Alternatively, we might expect a more linear relationship between secondary sexual trait expression and mating success. For example, in common yellowthroats, extra pair paternity increases approximately linearly with male mask size (Thusius et al. 2001). Where mating success is partly determined by factors independent of the secondary sexual trait (such as scramble competition or alternative mating tactics), we expect diminishing returns from investment in the trait, corresponding to a decelerating power function. Lastly, if there is a threshold of secondary sexual trait size that dramatically increases mating success, subject to diminishing returns at very large trait sizes, this will result in a logistic function. Although we believe that these function shapes are biologically plausible, the relationship between secondary sexual trait size and reproductive success remains poorly known, and more empirical research in species with varying mating systems is required to confirm these assumptions of our model.

On the other hand, the relationship between investment in somatic maintenance and lifespan is likely to be much less variable. Because return on investment in somatic maintenance in terms of increased lifespan is associated with internal physiological processes such as increasing efficiency of cellular maintenance, we expect a function with diminishing returns. Therefore, we assume a function form representing diminishing lifespan returns on investment in somatic maintenance:

(5) $$\begin{array}{c} E=\omega^{p}\\ ∴E={\left(\mathrm{qT}\right)}^{p} \end{array}$$

where 0 < *p* < 1. This curve passes through the points (0,0) and (1,1) meaning that an individual that invests nothing in somatic maintenance has zero lifespan, and the maximum possible lifespan is 1. A linear relationship between investment in somatic maintenance and lifespan is also plausible, but using this function form leads to qualitatively similar results (see Supplementary Materials, Figs. S1 and S2, for effects of varying *p*).

## ANALYSIS

Functions for mating success and fitness used in our models are outlined in Table 2. To investigate linear, accelerating power, and decelerating power function shapes, the general “power” model in Table 1 was initially parameterized as follows: for the linear model, *n* = 1; for the decelerating power model, *n* = 1/3; and for the accelerating power model, *n* = 3. For the tan function, *m* and *r* are set at 1.5 and 14.101, respectively so that this function is the same shape as the one used by Kokko (2001), but is constrained to the same limits as the other functions used in our analysis. In the logistic model, the constants *x, y*, and z are set at 1, 12, and 0.5, respectively so that the function asymptotes to zero and one at its’ minimum and maximum, respectively. The other functions pass through (0,0) and (1,1), meaning that *b* is zero if males invest nothing in the secondary sexual trait, and the maximum value of *b* is 1 for values 0 < *T* < 1 (Fig. 1). We also initially assume that males that do not express the secondary sexual trait achieve no matings (i.e., *s* = 0). Therefore, for the values of *T* that we are interested in, the *b*(*γ*) functions only differ in shape, but do not differ in the minimum or maximum expected mating success (Fig. 1), hence any differences in optimal investment arise due to differences in marginal benefits, rather than differences in absolute minimum or maximum values. We assume throughout that lifespan is a saturating function of investment in somatic maintenance (*p* = 1/3). Numerically solving each *W*(*q,T*) equation for q^*^ yields a function *q* ^*^(*T*), which represents how q^*^ changes with condition over the range 0 < *T* < 1. This allows us to find the optimal lifespan, E^*^, over the same values of *T* using equation (5).

**Table 2:** Models of fitness based on different hypothetical relationships between investment in a secondary sexual trait and mating success

*f*(γ)	*E*	*b*	*W*
Power			
Linear: *n* = 1 Decelerating power: 0 < *n* < 1 Accelerating power: *n* > 1	${\left(qT\right)}^{p}$	${\left((1-q)T\right)}^{n}+s$	${\left(qT\right)}^{p}*\left({\left((1-q)T\right)}^{n}+s\right)$
Logistic	${\left(qT\right)}^{p}$	$\frac{x}{1+e^{-y((1-q)T-z)}}+s$	${\left(qT\right)}^{p}*\left(\frac{x}{1+e^{-y((1-q)T-z)}}+s\right)$
Tan	${\left(qT\right)}^{p}$	$\frac{\tan(m((1-q)T))}{r}+s$	${\left(qT\right)}^{p}*\left(\frac{\tan(m((1-q)T))}{r}\right)+s$

**Figure 2:**
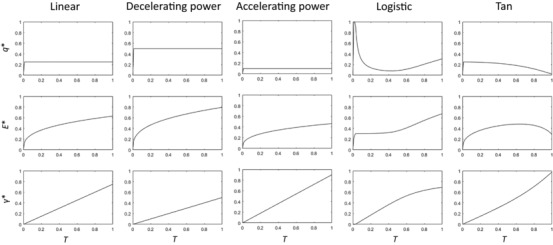
Optimal allocation to somatic maintenance, *q* ^*^, and resulting optimal lifespan, E^*^, and secondary sexual trait expression, *γ* ^*^, as a function of condition, *T*, for the models outlined in Table 2 and in text, with s=0.

Figure 2 shows the relationship between condition, *T*, and optimal allocation to somatic maintenance (*q* ^*^), and optimal lifespan (E^*^) for each of the fitness functions representing different relationships between secondary sexual trait expression and mating success (Table 2). In agreement with the findings of Kokko (2001), these results show that the shape of the mating success curve can affect the patterns of condition‐dependent investment in somatic maintenance. Generally, there is a positive relationship between condition and lifespan, except when mating success increases very sharply at high levels of investment in a secondary sexual trait, as in the tan function, where there is a negative correlation at high values of condition. For all forms of the power function that we examined, including the linear function, q^*^ is constant over all values of condition, but varies depending on whether the value of *n* is greater than or less than 1. This results in a positive correlation between condition and lifespan for each of these functions. Under the logistic model, there is no relationship between condition and optimal lifespan at low values of condition, but at higher values of condition lifespan increases rapidly with increasing condition.

Figure 2 also shows the optimal allocation to secondary sexual trait expression, *γ* ^*^, for each model. In each case, there is an overall positive relationship between condition and secondary sexual trait expression, although under the logistic model the slope of this relationship declines at high values of condition.

## THE EFFECT OF MINIMUM MATING SUCCESS

In the preceding analysis, we assumed that minimum mating success is zero when investment in the secondary sexual trait is zero (i.e., *s* = 0), but this is probably not the case in many mating systems. Low‐condition males of many species are able to achieve matings in ways that do not involve the use of the signal or weapon traits employed by high‐condition males (Gross 1996). For example, subordinate males of many species resort to sneaker tactics, including horned beetles (Emlen 1997), amphipods (Clark 1997), and peacock blennies (Gonçalves et al. 2003). Minimum expected mating success might also be greater than zero for males that do not express the secondary sexual trait if there is a high element of chance to mating. For example, under scramble competition, males may be able to gain some matings just by being around females long enough. Low‐condition males of some species (e.g., some horned beetles: Emlen 1994) can suppress secondary sexual trait expression almost completely; by contrast, in other species (such as some cervids: Suttie and Hamilton 1983), even males in low condition still express the secondary sexual trait to some degree, perhaps as a result of developmental constraints. However, regardless of the minimum possible level of secondary sexual trait expression, γ = 0 represents the minimum biologically possible level of investment in the secondary sexual trait in our models. When *s* > 0, the function relating male mating success to investment in the secondary sexual trait does not pass through the origin: that is, when γ = 0, *b* > 0.

We therefore asked how the ability to achieve matings without investment in the secondary sexual trait affects optimal *q* across the range of condition. To address this question, we adjusted the same models for mating success as outlined above by setting the constant, *s*, to a positive value (i.e., *s* > 0; Table 2). The maximum mating success therefore becomes 1 + *s*, and the minimum mating success (when γ = 0) becomes *b* = *s* for each model. For the following analyses *s* = 0.1. Varying *s* over a wide range of values does not qualitatively change patterns of optimal q^*^ and E^*^, except in some cases at very high values of *s* that select for decreased investment in secondary sexual traits (see Supplementary materials Fig. S3).

**Figure 3:**
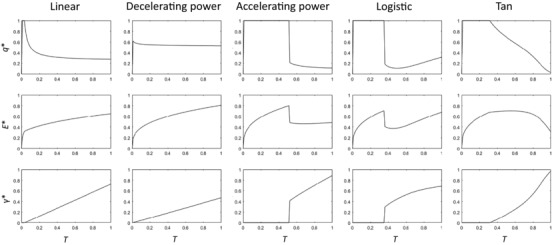
Optimal allocation to somatic maintenance, *q* ^*^, and resulting optimal lifespan, *E* ^*^, and secondary sexual trait expression, *Y* ^*^, as a function of condition, *T*, for the models outlined in Table 2 and in text, with *s*=0.1.

Figure 3 shows the relationship between *T* and *q* ^*^, and the subsequent optimal lifespan (E^*^) and optimal secondary sexual trait expression (γ^*^) for the models shown in Table 2 where *s* = 0.1. With the accelerating power, tan, and logistic functions, low‐condition individuals are selected to allocate all of their resources to somatic maintenance, and invest nothing in the secondary sexual trait. In the tan function, this results in a similar overall relationship as where *s* = 0: there is a positive relationship between condition and lifespan at low values of condition, but a negative relationship at high values of condition. However, in the accelerating power and logistic models, the patterns of optimal allocation indicate that there are two distinct optimal life‐history strategies within the population. While low‐condition individuals invest in extending their lifespan, high‐condition individuals are selected to increase investment in the secondary sexual trait. This results in a negative correlation between condition and lifespan across the specified range of condition. On the other hand, when payoffs from investing in a secondary sexual trait increase linearly, or have a saturating response (decelerating power function), optimal allocation patterns do not change qualitatively with changing values of *s*: there is always a positive correlation between condition and lifespan.

**Figure 4:**
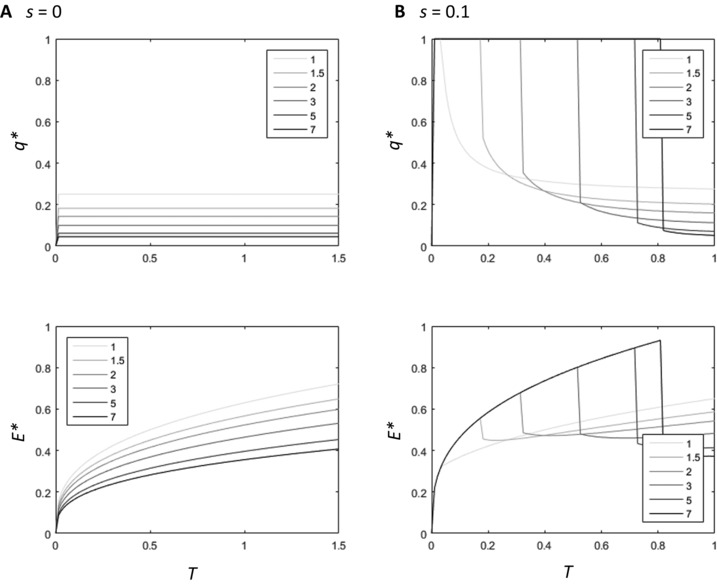
The change in q^*^ and *E* ^*^ with increasing condition for six fitness functions where the relationship between secondary sexual trait expression and mating success is an accelerating power function, where *s* = 0 (A), and *s* = 0.1 (B). The value of *n* for each function is shown in the legend.

**Figure 5:**
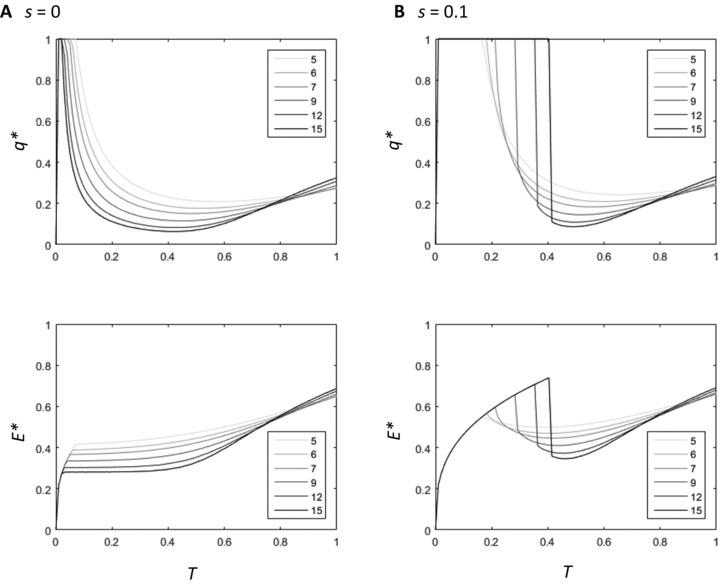
The change in q^*^ and *E* ^*^ with increasing condition for six fitness functions where the relationship between secondary sexual trait expression and mating success is logistic, where *s* = 0 (A), and *s* = 0.1 (B). The value of *y* for each function is shown in the legend.

Our results suggest that a negative correlation between condition and lifespan emerges only when the payoffs from investing in the secondary sexual trait increase nonlinearly with increasing investment. Thus, we further investigated how patterns of nonlinearity affect evolution of optimal lifespan for the accelerating power and logistic functions (Figs. 4 and 5). When *s* = 0, increasing nonlinearity in both the accelerating power function model (increasing *n* parameter, Fig. 4) and the logistic function model (increasing the slope, *y*, parameter, Fig. 5) selects for increased investment in the secondary sexual trait over all values of *T* (Figs. 4A, 5A). In these cases, low‐condition individuals are never selected to decrease investment in the secondary sexual trait to increase life span, because this would reduce their mating success to zero. On the other hand, a small increase in secondary sexual trait expression has a significant effect on overall fitness because the relative increase in mating success is high for both low‐ and high‐condition individuals. Although this pattern changes at very high values of *T* in the logistic function, where the marginal benefits for investing in a secondary sexual trait begin to decrease (Fig. 5A), the overall affect is that increasing nonlinearity does not affect the overall positive correlation between condition and lifespan. Hence, the allocation patterns from the tan function appear to be specific to this function shape: increasing the steepness of the payoff curve does not select for a negative relationship between condition and lifespan in other function shapes we used (table 2).

By contrast, when minimum mating success is greater than zero (*s* > 0), the optimal allocation patterns for low‐condition individuals change. In the high‐condition individuals, increasing nonlinearity still selects for increased investment in the secondary sexual trait, resulting in a similar investment pattern for this region of condition as in models where *s* = 0. However, the marginal benefits of investing in a secondary sexual trait decrease for low‐condition individuals. The overall effect is that increasing nonlinearity of mating success selects for high‐condition individuals to increase investment in *γ*, but for low‐condition individuals to decrease their investment in *γ* to zero and instead extend their lifespan. This results in greater discontinuity in patterns of optimal lifespan and secondary sexual trait expression, as low and high‐condition males gain higher fitness with increasingly different investment strategies. As a result, as *n* increases, the relationship between condition and optimal lifespan becomes more negative across the range of condition. The patterns in Figs. 4B and 5B indicate that it is this selection on low‐condition individuals that drives the overall pattern of relatively shorter lifespans in high‐condition individuals, rather than solely selection on high‐condition individuals to sacrifice investment in lifespan to increase investment in reproduction.

We also investigated the consequences of adjusting the value of *n* in the decelerating power model, to make mating success payoffs more or less similar to a linear model. For the decelerating power model, the positive relationship between lifespan and condition persists regardless of the value of the exponent both when *s* = 0 and when *s* = 0.1 (Supplementary materials, Fig. S4).

## Discussion

Our models have uncovered two different scenarios where we expect a negative relationship between condition and lifespan to evolve. The first scenario involves accelerating marginal benefits to investment in a secondary sexual trait. In line with Kokko (2001), we find that a rapidly accelerating relationship between investment in secondary sexual trait expression and mating success (the tan function) can select for high‐condition individuals to decrease investment in survival. Also in agreement with Kokko (2001), we found that selection for reduced investment in lifespan is not a general feature of all functions with a similar shape to the tan function. The second scenario occurs when investment in secondary sexual traits is not the only possible route to mating, such that males that invest minimally in secondary sexual traits still achieve nonzero average mating success. We find that, in such cases, a negative relationship between condition and lifespan also emerges because low‐condition males are selected to invest exclusively in lifespan. Hence, we show that selection on low‐condition individuals is also important to consider when investigating patterns of condition‐dependent ageing. These findings predict the patterns of sexual selection and types of mating systems that could select for different patterns of condition‐dependent ageing observed in empirical studies, and could lead to an enhanced understanding of variation in the condition‐dependence of lifespan and ageing both within and between species.

As shown by Kokko (2001), when the payoffs from investing in a secondary sexual trait increase at an accelerating rate with increasing investment in the trait (represented by a tan function), males in high condition are selected to decrease investment in longevity, so that at higher levels of condition there is a negative relationship between condition and lifespan. A tan function represents a case where, at high levels of investment, marginal benefits of investment in secondary sexual trait expression increase at an increasing rate. This may represent a biological system where the males that invest heavily in traits that confer an advantage in male–male competition are very effective at monopolising females, such as in mandrills (Setchell et al. 2005) and elephant seals (Le Boeuf 1974). Hence, in these systems, if there are no resource‐limited individuals within the population and little variation in resource acquisition between individuals, then we expect a negative relationship between condition and lifespan to evolve.

Alternatively, a negative relationship between condition and lifespan can evolve when low‐condition individuals are selected to increase investment in lifespan at the cost of secondary sexual trait expression. This occurs where the payoffs from investing in a secondary sexual trait are the highest for high‐condition individuals and much lower for low‐condition individuals (shown in our accelerating power and logistic models), and when minimum expected mating success is greater than zero. In these cases, low and high‐condition males pursue different allocation strategies, which results in a discontinuous allocation pattern (rather than a gradual decrease in investment in lifespan, such as that observed with the tan functions). The population might therefore consist of longer lived sneaker males, and shorter lived males with large secondary sexual traits. Interestingly, the patterns of secondary sexual trait expression obtained with these models approximate observed patterns of male secondary sexual trait (horn) expression in some species of beetles that show sigmoidal trait scaling and condition‐dependent male mating strategies, including alternative/sneak tactics in low‐condition males (e.g., Emlen 1994, 1997). Hence, our analysis suggests that nonlinear payoffs from investment in reproduction could favor the evolution of two distinct condition‐dependent male life‐history strategies within the population. Our results also highlight the general importance of considering minimum mating success, a factor that could have important consequences for the evolution of both mating and life‐history strategies. In a model similar to the one used here, nonzero minimum mating success was shown to promote the evolution of positive allometry of secondary sexual traits (Fromhage and Kokko 2014). Our analysis shows that nonzero minimum mating success could also have important consequences for the evolution of condition‐dependent ageing.

Where the marginal benefits from increasing investment in a secondary sexual trait are similar for large and small trait sizes (linear model), or higher for small trait sizes (decelerating power model), positive associations between condition and lifespan are predicted regardless of whether matings can be achieved without expressing the secondary sexual trait. These findings are consistent with previous theoretical studies including Nur and Hasson (1984). Data from common yellowthroats may fit these predictions. In this socially monogamous species, male mating success is correlated with mask size, males achieving more extra pair copulations with increasing mask size, and extra pair matings increase approximately linearly with mask size (Thusius et al. 2001). As such, the finding that larger mask size is associated with increased survival probability (Dunn et al. 2013) fits the predictions of our model.

Our findings reflect the key assumption that the payoffs from investment in lifespan do not increase steeply in the way that payoffs from investment in reproduction do. We also assume that the mating success function is highly variable between populations, and can take on multiple forms, while the relationship between investment in somatic maintenance and lifespan is less variable between populations. We believe that this is a reasonable assumption because of the nature of the physiological systems that determine ageing rate, and the fact that these systems are unlikely to vary between species to the same extent as the traits that determine mating success. We also assumed that the costs of secondary sexual traits arise only through resource allocation trade‐offs. However, secondary sexual traits can also impose other types of costs. For example, increased secondary sexual trait expression can result in increased predation pressure (Zuk and Kolluru 1998), physiological costs such as increased telomere erosion rates (Giraudeau et al. 2016), or reduced immunocompetence (Faivre et al. 2003). Including other types of costs in the model may further reduce the expected lifespan of males with high investment in secondary sexual traits.

Our model assumes that resource allocation decisions are fixed at the onset of reproduction. This assumption is reasonable for systems such as holometabolous insects that invest in expression of morphological secondary sexual traits during juvenile development, and cannot alter this investment as adults. However, in long‐lived, iteroparous species where investment can be adjusted at each breeding season (such as in male cervids that regrow antlers each year, or birds that invest in nuptial plumage at the start of each breeding season), optimal investment strategies may change with age or in response to somatic wear‐and‐tear. Such facultative adjustment is also likely to characterize investment in costly sexual behavior. Such systems and traits may be characterized by feedback between secondary sexual trait expression and resulting somatic wear‐and‐tear, such that individuals might be selected to dynamically adjust their investment in secondary sexual traits and in somatic maintenance so as to maximize expected lifetime reproductive success. Understanding the evolution of condition‐dependent ageing in such systems may require the development of more complex models.

Our results reveal a wide variety of shapes in the relationship between condition and optimal lifespan. For example, in the tan function there is an inverted‐U shape when the range of condition encompasses both the relatively flat portion of the curve as well as the quickly accelerating portion of the curve. In the logistic model where *s* = 0 (males cannot achieve matings without investing in the secondary sexual trait), there is only a very weak positive relationship between condition and lifespan at low levels of condition, but a positive relationship at higher levels of condition. Additionally, with the logistic model where *s* > 0, our analysis predicts that individuals with intermediate values of *T* will have the shortest lifespans, because those intermediate individuals have the most to gain from sacrificing investment in lifespan for the sake of enhancing secondary sexual traits. These patterns suggest that an empirical study of condition‐dependent ageing could find any correlation between condition and lifespan – positive, negative, or neutral – depending on how the range of conditions investigated relates to the curves that define mating success and lifespan. Hence, future empirical studies of condition‐dependent ageing would benefit from including more than just two condition values or treatments in their analyses (as is frequently done, see Cotton et al. (2004) for a review) to reduce the risk of obtaining misleading patterns.

Our model can also provide insight into differences between the sexes in condition‐dependent ageing. In many species, males benefit more than females by sacrificing longevity for reproductive opportunities. This is because males are selected to pursue strategies involving high risk and immediate fitness pay‐offs (i.e., mating opportunities), whereas females are selected to pursue strategies involving less risky strategies with delayed payoff (i.e., resource accumulation and offspring production) (Vinogradov 1998; Bonduriansky et al. 2008; Adler and Bonduriansky 2014). In most species, females with the highest investment in reproduction are unlikely to be able to monopolize limiting resources in the same way that males can do. Thus, in females, the payoff from investment in reproductive traits, such as ovary size, egg size, or clutch size, is most likely to be linear or decelerating, while accelerating payoff function shapes are unlikely. Given that the only models in our study to predict a negative relationship between condition and lifespan featured increasing marginal benefits to increasing investment in reproduction, the fact that females rarely show a negative relationship between condition and lifespan may therefore be explained by the fact that female reproductive success rarely increases nonlinearly with increasing investment.

Whist empirical observations are broadly consistent with our predictions, we have not been able to find empirical studies reporting all the information required to fully test our models. In particular, there is a lack of studies that have quantified the relationship between secondary sexual trait expression and mating success in sufficient detail. Additionally, estimates of pay‐off functions for investment in somatic maintenance, as well as experimental studies investigating the relationship between condition and lifespan, are needed to test the predictions of our models.

## Conclusions

We show how variation in mating success payoffs from investment in a secondary sexual trait could shape condition‐dependent ageing patterns. Our results suggest that negative relationships between overall condition and survival or lifespan do not necessarily arise because of selection on high‐condition individuals to decrease investment in somatic maintenance. Under particular circumstances, low‐condition individuals could be selected to increase investment in somatic maintenance, and the evolution of investment strategies in low‐condition males can thereby potentially determine patterns of condition‐dependent ageing. In particular, we find that, when low‐condition individuals can gain matings despite minimal investment in secondary sexual trait expression, low‐condition individuals are selected to invest all their resources in extending their lifespan. Hence, we demonstrate the importance of considering the strategies of both low‐ and high‐condition individuals when investigating the evolution of condition‐dependent ageing patterns. Our findings have implications for both the evolution of condition‐dependent ageing and the evolution of condition‐dependent life‐history strategies. While our predictions are consistent with evidence from several species, further empirical research is needed to test these predictions.

Associate Editor: A. Charmantier

## Supporting information


**Figure S1**. The change in q^*^ and *E* ^*^ with increasing condition for each model investigated for six different potential relationships between somatic maintenance and lifespan, where *s* = 0. In each case *p* is given in the legend.
**Figure S2**. The change in q^*^ and *E* ^*^ with increasing condition for each model investigated for six different potential relationships between somatic maintenance and lifespan, where *s* = 0.1. In each case *p* is given in the legend.
**Figure S3**. The change in q^*^ and E^*^ with increasing condition for each model investigated for six different potential values of *s*. In each case *s* is given in the legend.
**Figure S4**. The change in q^*^ and *E* ^*^ with increasing condition for six fitness functions where the relationship between secondary sexual trait expression and mating success is a decelerating power function, where *s* = 0 (A), and *s* = 0.1 (B). The value of *m* for each function is shown in the legend.

## References

[evl336-bib-0001] Adler, M. I. , and R. Bonduriansky . 2014 Sexual conflict, life span, and aging. Cold Spring Harb. Perspect. Biol. 6:a017566. 24938876 10.1101/cshperspect.a017566 PMC4107991

[evl336-bib-0002] Andersson, M. 1982 Sexual selection, natural selection and quality advertisement. Biol. J. Linn. Soc. 17:375–393.

[evl336-bib-0003] Bérubé, C. H. , M. Festa‐Bianchet , and J. T. Jorgenson . 1999 Individual differences, longevity, and reproductive senescence in bighorn ewes. Ecology 80:2555–2565.

[evl336-bib-0004] Boggs, C. L. 2009 Understanding insect life histories and senescence through a resource allocation lens. Funct. Ecol. 23:27–37.

[evl336-bib-0005] Bonduriansky, R . 2007 The evolution of condition‐dependent sexual dimorphism. Am. Nat. 169:9–19. 17206580 10.1086/510214

[evl336-bib-0006] Bonduriansky, R. , A. Maklakov , F. Zajitschek , and R. Brooks . 2008 Sexual selection, sexual conflict and the evolution of ageing and life span. Funct. Ecol. 22:443–453.

[evl336-bib-0007] Bouwhuis, S. , B. C. Sheldon , S. Verhulst , and A. Charmantier . 2009 Great tits growing old: selective disappearance and the partitioning of senescence to stages within the breeding cycle. Proc. R Soc. B 276:2769–3277. 10.1098/rspb.2009.0457 PMC2839957 19403537

[evl336-bib-0008] Bouwhuis, S. , R. Choquet , B. C. Sheldon , and S. Verhulst . 2012 The forms and fitness cost of senescence: age‐specific recapture, survival, reproduction, and reproductive value in a wild bird population. Am. Nat. 179:E15–E27. 22173469 10.1086/663194

[evl336-bib-0009] Clark, R. A. 1997 Dimorphic males display alternative reproductive strategies in the marine amphipod *Jassa marmorata* holmes (Corophioidea: Ischyroceridae). Ethology 103:531–553.

[evl336-bib-0010] Cotton, S. , K. Fowler , and A. Pomiankowski . 2004 Do sexual ornaments demonstrate heightened condition‐dependent expression as predicted by the handicap hypothesis? Proc. R Soc. B 271:771–783. 10.1098/rspb.2004.2688 PMC1691662 15255094

[evl336-bib-0011] Davison, R. , C. L. Boggs , and A. Baudisch . 2014 Resource allocation as a driver of senescence: life history tradeoffs produce age patterns of mortality. J. Theor. Biol. 360:251–262. 25051533 10.1016/j.jtbi.2014.07.015

[evl336-bib-0012] Descamps, S. , J. M. Gaillard , S. Hamel , and N. G. Yoccoz . 2016 When relative allocation depends on total resource acquisition: implication for the analysis of trade‐offs. J. Evol. Biol. 29:1860–1866. 27200492 10.1111/jeb.12901

[evl336-bib-0013] Dunn, P. O. , J. L. Bollmer , C. R. Freeman‐Gallant , and L. A. Whittingham . 2013 MHC variation is related to a sexually selected ornament, survival, and parasite resistance in common yellowthroats. Evolution 67:679–687. 23461319 10.1111/j.1558-5646.2012.01799.x

[evl336-bib-0014] Emlen, D. J. 1994 Environmental control of horn length dimorphism in the beetle *Onthophagus acuminatus* (Coleoptera: Scarabaeidae). P. R. Soc. Lond. B 256:131–136.

[evl336-bib-0015] Emlen, D. J. 1997 Alternative reproductive tactics and male‐dimorphism in the horned beetle *Onthophagus acuminatus* (Coleoptera: Scarabaeidae). Behav. Ecol. Sociobiol. 41:335–341.

[evl336-bib-0016] Evans, M. R. 2003 Survival of male scarlet‐tufted malachite sunbirds (*Nectarinia johnstoni*) on Mount Kenya and the influence of ornamentation. Biol. J. Linn. Soc. 80:125–133.

[evl336-bib-0017] Faivre, B. , A. Grégoire , M. Préault , F. Cézilly , and G. Sorci . 2003 Immune activation rapidly mirrored in a secondary sexual trait. Science 300:103. 12677062 10.1126/science.1081802

[evl336-bib-0018] Fromhage, L. , and H. Kokko . 2014 Sexually selected traits evolve positive allometry when some matings occur irresepective of the trait. Evolution 68:1332–1338. 24410284 10.1111/evo.12349

[evl336-bib-0019] Gonçalves, D. , T. Fagundes , and R. Oliveira . 2003 Reproductive behaviour of sneaker males of the peacock blenny. J. Fish Biol. 63:528–532.

[evl336-bib-0020] Giraudeau, M. , C. R. Friesen , J. Sudyka , N. Rollings , C. M. Whittington , M. R. Wilson , and M. Olsson . 2016 Ageing and the cost of maintaining coloration in the Australian painted dragon. Biol. Lett. 12:20160077. 27405377 10.1098/rsbl.2016.0077 PMC4971163

[evl336-bib-0021] Gross, M. R. 1996 Alternative reproductive strategies and tactics: diversity within sexes. Trends Ecol. Evol. 11:92–98. 21237769 10.1016/0169-5347(96)81050-0

[evl336-bib-0022] Hill, G. E. 2011 Condition‐dependent traits as signals of the functionality of vital cellular processes. Ecol. Lett. 7:625–634. 10.1111/j.1461-0248.2011.01622.x 21518211

[evl336-bib-0023] Hooper, A. K. , F. Spagopoulou , Z. Wylde , A. A. Maklakov , and R. Bonduriansky . 2017 Ontogenetic timing as a condition‐dependent life history trait: high‐condition males develop quickly, peak early, and age fast. Evolution 71:671–685. 28067402 10.1111/evo.13172

[evl336-bib-0024] Hughes, K. A. , and R. M. Reynolds . 2005 Evolutionary and mechanistic theories of ageing. Annu. Rev. Entomol. 50:421–445. 15355246 10.1146/annurev.ento.50.071803.130409

[evl336-bib-0025] Hunt, J. , R. Brooks , M. D. Jennions , M. J. Smith , C. L. Bentsen , and L. F. Bussiere . 2004 High‐quality male field crickets invest heavily in sexual display but die young. Nature 432:1024–1027. 15616562 10.1038/nature03084

[evl336-bib-0026] Jones, O. R. , A. Scheuerlein , R. Salguero‐Gomez , C. G. Camarda , R. Schaible , B. B. Casper , J. P. Dahlgren , J. Ehrlen , M. B. Garcia , E. S. Menges , et al. 2014 Diversity of ageing across the tree of life. Nature 505:169–173. 24317695 10.1038/nature12789 PMC4157354

[evl336-bib-0027] Judge, K. A. , J. J. Ting , D. T. Gwynne , and N. Wedell . 2008 Condition dependence of male life span and calling effort in a field cricket. Evolution 62:868–878. 18194475 10.1111/j.1558-5646.2008.00318.x

[evl336-bib-0028] Kirkwood, T. B. L. 1977 Evolution of ageing. Nature 270:301–304. 593350 10.1038/270301a0

[evl336-bib-0029] Kirkwood, T. B. L. , and M. R. Rose . 1991 Evolution of senescence: late survival sacrificed for reproduction. Philos. T. Roy. Soc. B 332:15–24. 10.1098/rstb.1991.0028 1677205

[evl336-bib-0030] Kokko, H. 1998 Good genes, old age and life‐history trade‐offs. Evol. Ecol. 12:739–750.

[evl336-bib-0031] Kokko, H. 2001 Fisherian and “good genes” benefits of mate choice: how (not) to distinguish between them. Ecol. Lett. 4:322–326.

[evl336-bib-0032] Kowald, A. , and T. Kirkwood . 2015 Evolutionary significance of ageing in the wild. Exp. Gerontol. 71:89–94. 26292149 10.1016/j.exger.2015.08.006

[evl336-bib-0033] Kruuk, L. E. B. , J. Slate , J. M. Pemberton , S. Brotherstone , F. Guinness , T. Clutton‐Brock , and D. Houle . 2002 Antler size in red deer: heritability and selection but no evolution. Evolution 56:1683–1695. 12353761 10.1111/j.0014-3820.2002.tb01480.x

[evl336-bib-0034] Le Boeuf, B. J. 1974 Male‐male competition and reproductive success in elephant seals. Am. Zool. 14:163–176.

[evl336-bib-0035] Lemaitre, J. F. , V. Berger , C. Bonenfant , M. Douhard , M. Gamelon , F. Plard , and J. M. Gaillard . 2015 Early‐late life trade‐offs and the evolution of ageing in the wild. Proc. R. Soc. B 282:20150209. 10.1098/rspb.2015.0209 PMC4426628 25833848

[evl336-bib-0036] Mangel, M. , and S. B. Munch . 2005 A life‐history perspective on short‐ and long‐term consequences of compensatory growth. Am. Nat. 166:E155–E176. 16475079 10.1086/444439

[evl336-bib-0037] McCleery, R. H. , C. M. Perrins , B. C. Sheldon , and A. Charmantier . 2008 Age‐specific reproduction in a long‐lived species: the combined effects of senescence and individual quality. Proc. R. Soc. B 275:963–970. 10.1098/rspb.2007.1418 PMC2599932 18230597

[evl336-bib-0038] Møller, A. P. , and T. Szép . 2002 Survival rate of adult barn swallows *Hirundo rustica* in relation to sexual selection and reproduction. Ecology 83:2220–2228.

[evl336-bib-0039] Munguía‐Steyer, R. , A. Córdoba‐Aguilar , and A. Romo‐Beltrán . 2010 Do individuals in better condition survive for longer? Field survival estimates according to male alternative reproductive tactics and sex. J. Evolution Biol. 23:175–184. 10.1111/j.1420-9101.2009.01894.x 20069722

[evl336-bib-0040] Nur, N. , and O. Hasson . 1984 Phenotypic plasticity and the handicap principle. J. Theor. Biol. 110:275–297.

[evl336-bib-0041] Preston, B. T. , M. S. Jamle , Y. Hingrat , F Lacroix , and G. Sorci . 2011 Sexually extravagant males age more rapidly. Ecol. Lett. 14:1017–1024. 21806745 10.1111/j.1461-0248.2011.01668.x

[evl336-bib-0042] Radwan, J. , and I. Bogacz . 2000 Comparison of life‐history traits of the two male morphs of the bulb mite, *Rhizoglyphus robini* . Exp. Appl. Acarol. 24:115–121. 11108391 10.1023/a:1006492903270

[evl336-bib-0043] Roff, D. A. , and D. J. Fairbairn . 2007 The evolution of trade‐offs: where are we? J. Evolution Biol. 20:433–447. 10.1111/j.1420-9101.2006.01255.x 17305809

[evl336-bib-0044] Rowe, L. , and D. Houle . 1996 The lek paradox and the capture of genetic variance by condition dependent traits. P. R. Soc. Lond. B 263:1415–1421.

[evl336-bib-0045] Saino, N. , A. M. Bolzern , and A. P. Møller . 1997 Immunocompetence, ornamentation, and viability of male barn swallows (*Hirundo rustica*). Proc. Natl. Acad. Sci. USA 94:549–552. 9012821 10.1073/pnas.94.2.549 PMC19550

[evl336-bib-0046] Setchell, J. M. , M. Charpentier , and E. J. Wickings . 2005 Mate guarding and paternity in mandrills: factors influencing alpha male monopoly. Anim. Behav. 70:1105–1120.

[evl336-bib-0047] Simmons, L. W. , S. Lüpold , and J. L. Fitzpatrick . 2017 Evolutionary trade‐off between secondary sexual traits and ejaculates. Trends Ecol. Evol. 32:964–976. 29050795 10.1016/j.tree.2017.09.011

[evl336-bib-0048] Simons, M. J. P. , M. Briga , E. Koetsier , R. Folkertsma , M. D. Wubs , C. Dijkstra , and S. Verhulst . 2012 Bill redness is positively associated with reproduction and survival in male and female zebra finches. PLOS One 7:e40721. 22808243 10.1371/journal.pone.0040721 PMC3395645

[evl336-bib-0049] Suttie, J. M. , and W. J. Hamilton . 1983 The effect of winter nutrition on growth of young Scottish Red deer (*Cervus elaphus*). J. Zool. 201:153–159.

[evl336-bib-0050] Thusius, K. J. , K. A. Peterson , P. O. Dunn , and L. A. Whittingham . 2001 Male mask size is correlated with mating success in the common yellowthroat. Anim. Behav. 62:435–446.

[evl336-bib-0051] van den Heuvel, J. , S. English , and T. Uller . 2016 Disposable soma theory and the evolution of maternal effects on ageing. PLOS One 11:e0145544. 26752635 10.1371/journal.pone.0145544 PMC4709080

[evl336-bib-0052] van Noordwijk, A. J. , and G. de Jong . 1986 Acquisition and allocation of resources: their influence on variation in life history tactics. Am. Nat. 128:137–142.

[evl336-bib-0053] Vinogradov, A. E. 1998 Male reproductive strategy and decreased longevity. Acta Biotheor. 46:157–160. 9691260 10.1023/a:1001181921303

[evl336-bib-0054] Zuk, M. , and G. R. Kolluru . 1998 Exploitation of sexual signals by predators and parasitoids. Q Rev. Biol. 73:415–438.

