# Supplementary material for: Sexual competition and the evolution of condition‐dependent ageing
Source: Evol Lett. 2018 Jan 13;2(1):37–48. doi: 10.1002/evl3.36 (PMC6089505; doi:10.1002/evl3.36)
Supplement: Supplementary file 1 — Figure S1. The change in q* and E * with increasing condition for each model investigated for six different potential relationships between somatic maintenance and lifespan, where s = 0. In each case p is given in the legend. Figure S2. The change in q* and E * with increasing condition for each model investigated for six different potential relationships between somatic maintenance and lifespan, where s = 0.1. In each case p is given in the legend. Figure S3. The change in q* and E* with increasing condition for each model investigated for six different potential values of s. In each case s is given in the legend. Figure S4. The change in q* and E * with increasing condition for six fitness functions where the relationship between secondary sexual trait expression and mating success is a decelerating power function, where s = 0 (A), and s = 0.1 (B). The value of m for each function is shown in the legend. [file EVL3-2-37-s001.pdf]

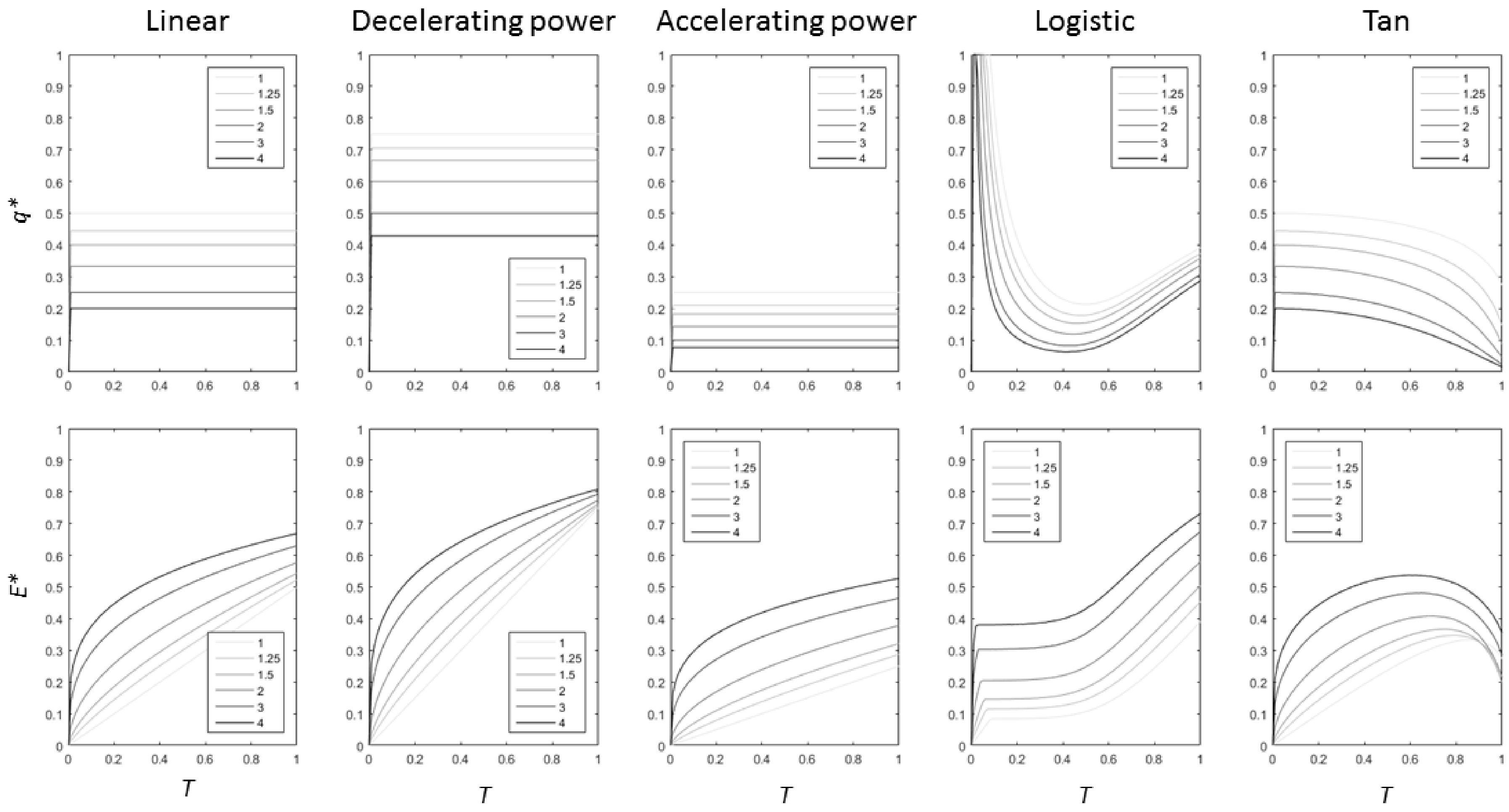

Fig. S1 The change in q^* and E^* with increasing condition for each model investigated for six different potential relationships between somatic maintenance and lifespan, where s=0. In each case p is given in the legend.

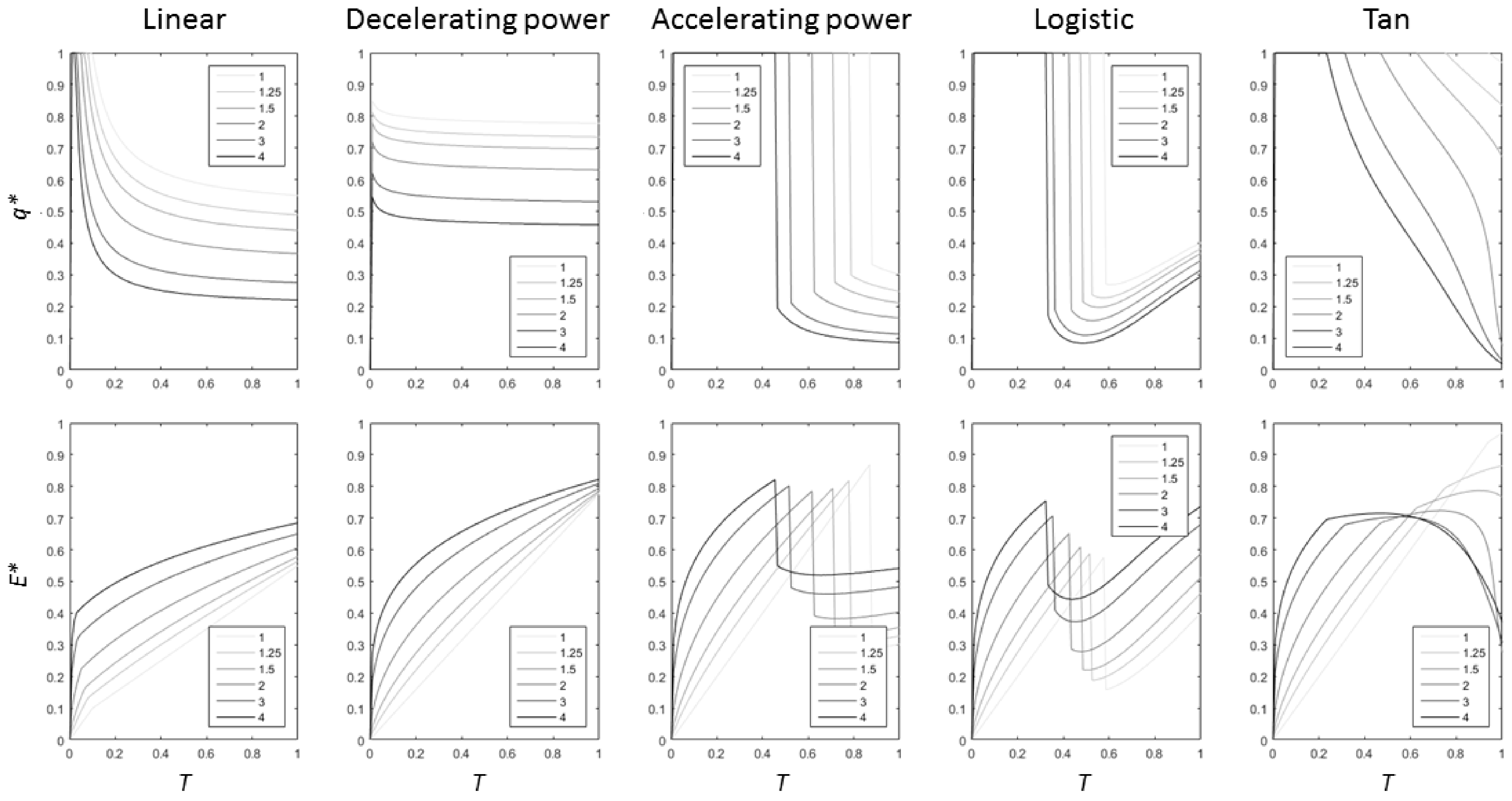

Fig. S2 The change in q^* and E^* with increasing condition for each model investigated for six different potential relationships between somatic maintenance and lifespan, where s=0.1. In each case p is given in the legend.

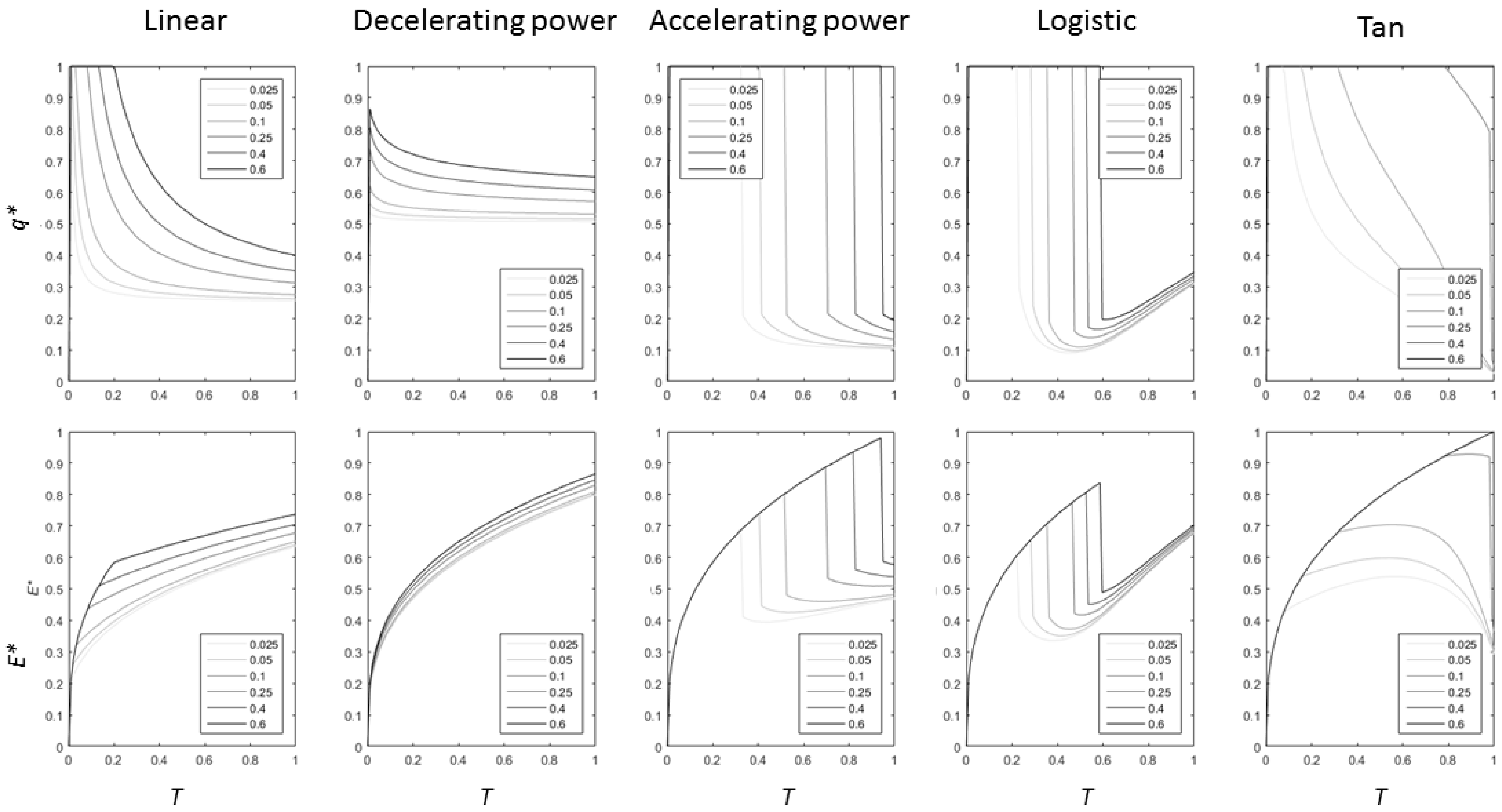

Fig. S3 The change in $q*$ and $E*$ with increasing condition for each model investigated for six different potential values of s. In each case s is given in the legend.

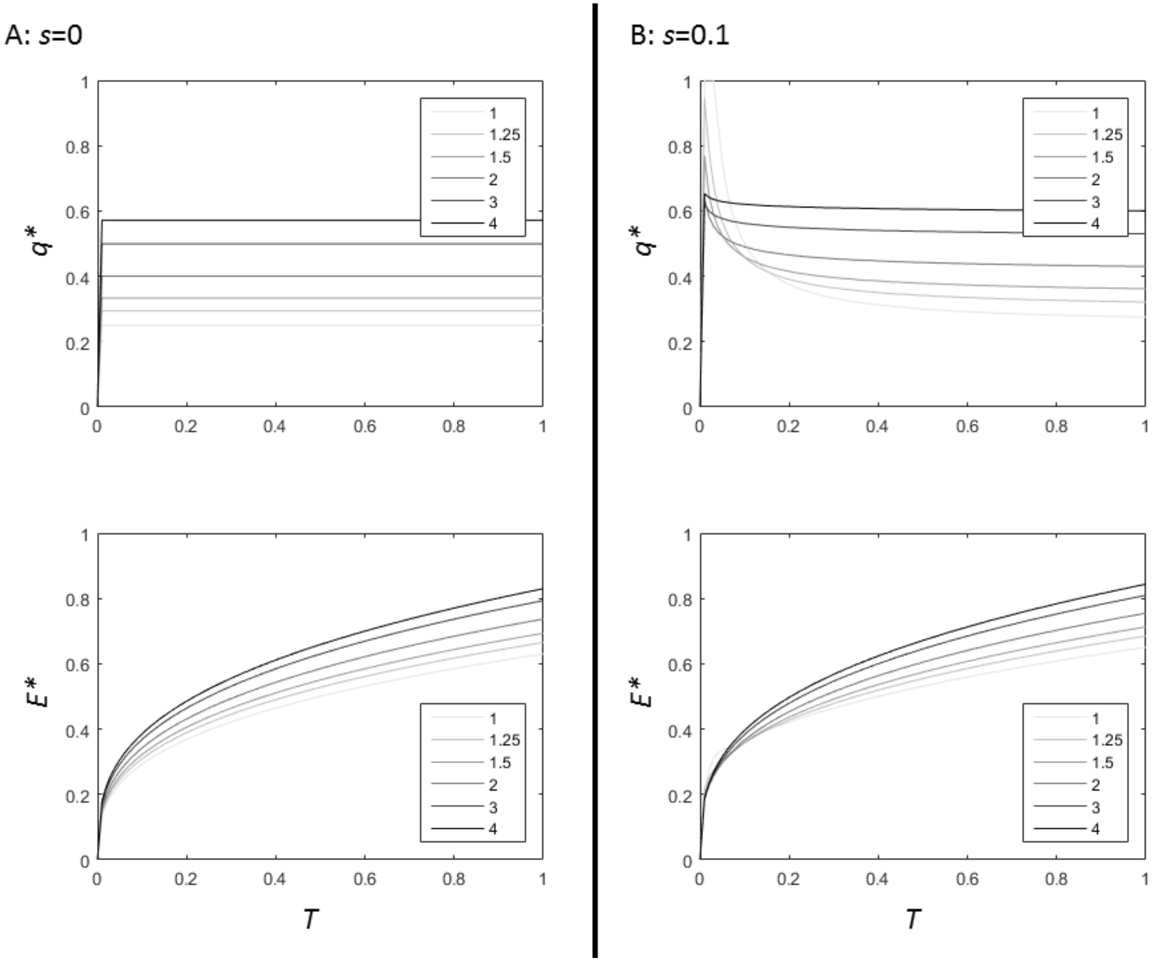

Fig S4. The change in q^* and E^* with increasing condition for six fitness functions where the relationship between secondary sexual trait expression and mating success is a decelerating power function, where s=0 (A), and s=0.1 (B). The value of m for each function is shown in the legend.
